# Complete Response to Pembrolizumab After Progression on Avelumab Maintenance in Metastatic Urothelial Carcinoma

**DOI:** 10.1002/iju5.70160

**Published:** 2026-03-08

**Authors:** Fumihiro Ito, Koki Kobayashi, Gaku Hayashi, Shunsuke Kamijo, Takashi Fujita

**Affiliations:** ^1^ Department of Urology Gifu Prefectural Tajimi Hospital Tajimi Japan

**Keywords:** avelumab, maintenance therapy, pembrolizumab, rechallenge, urothelial carcinoma

## Abstract

**Introduction:**

Avelumab maintenance after response to platinum chemotherapy improves outcomes in metastatic urothelial carcinoma. Progression during maintenance presents a therapeutic dilemma.

**Case Presentation:**

A 72‐year‐old man underwent radical cystectomy and left nephroureterectomy for high‐grade urothelial carcinoma. Two and a half years later, a solitary pelvic nodal recurrence achieved complete response to gemcitabine–cisplatin, after which avelumab maintenance was started. After 16 cycles, the same node regrew, indicating progression. Pembrolizumab was initiated and induced a second complete response after four cycles. He remains recurrence‐free for three years without immune‐related adverse events.

**Conclusion:**

Durable remission with pembrolizumab following progression on avelumab suggests that switching from PD‐L1 to PD‐1 blockade can overcome resistance in selected patients; prospective studies and biomarker strategies, including PD‐L2 assessment, are warranted and may guide individualized rechallenge strategies.

**Keynote Message**

Sequential PD‐L1 and PD‐1 blockade can induce durable remission in selected patients with metastatic urothelial carcinoma, highlighting the potential to overcome resistance and the need for biomarker‐guided strategies such as PD‐L2 assessment.

**Abbreviations**
CR: complete response
CT: computed tomography
ECOG: Eastern Cooperative Oncology Group
eGFR: estimated glomerular filtration rate
EV: Enfortumab vedotin
ICI: immune checkpoint inhibitor
mUC: metastatic urothelial carcinoma
PD: progressive disease
PD‐1: programmed cell death‐1
PD‐L1: programmed cell death ligand‐1
PD‐L2: programmed cell death ligand‐2
PS: performance status
SD: stable disease
UC: urothelial carcinoma

## 1 Introduction

Avelumab first‐line maintenance after response or stable disease with platinum‐based chemotherapy improves overall survival in advanced urothelial carcinoma [1, 2, 3] and is recommended by contemporary guidelines [2]. Nevertheless, a substantial proportion of patients progress during or after maintenance, and optimal post‐progression sequencing remains unsettled. Available options include enfortumab vedotin after prior platinum and an immune checkpoint inhibitor, which improved survival in EV‐301 [4], and the combination of enfortumab vedotin plus pembrolizumab, which has redefined the first‐line standard of care (EV‐302/KEYNOTE‐A39) [5]. Whether switching from a PD‐L1 antibody to a PD‐1 antibody can recapture antitumor activity without intervening chemotherapy is unclear and understudied [6, 7]. We report a durable complete response to pembrolizumab after progression during avelumab maintenance.

## 2 Case Presentation

A 72‐year‐old man with hypertension and a 45 pack‐year smoking history underwent radical cystectomy, left nephroureterectomy, and cutaneous ureterostomy for high‐grade urothelial carcinoma.

Clinical staging (preoperative imaging): cT2–3N0M0 (AJCC/UICC TNM, 8th edition).

**FIGURE 1:**
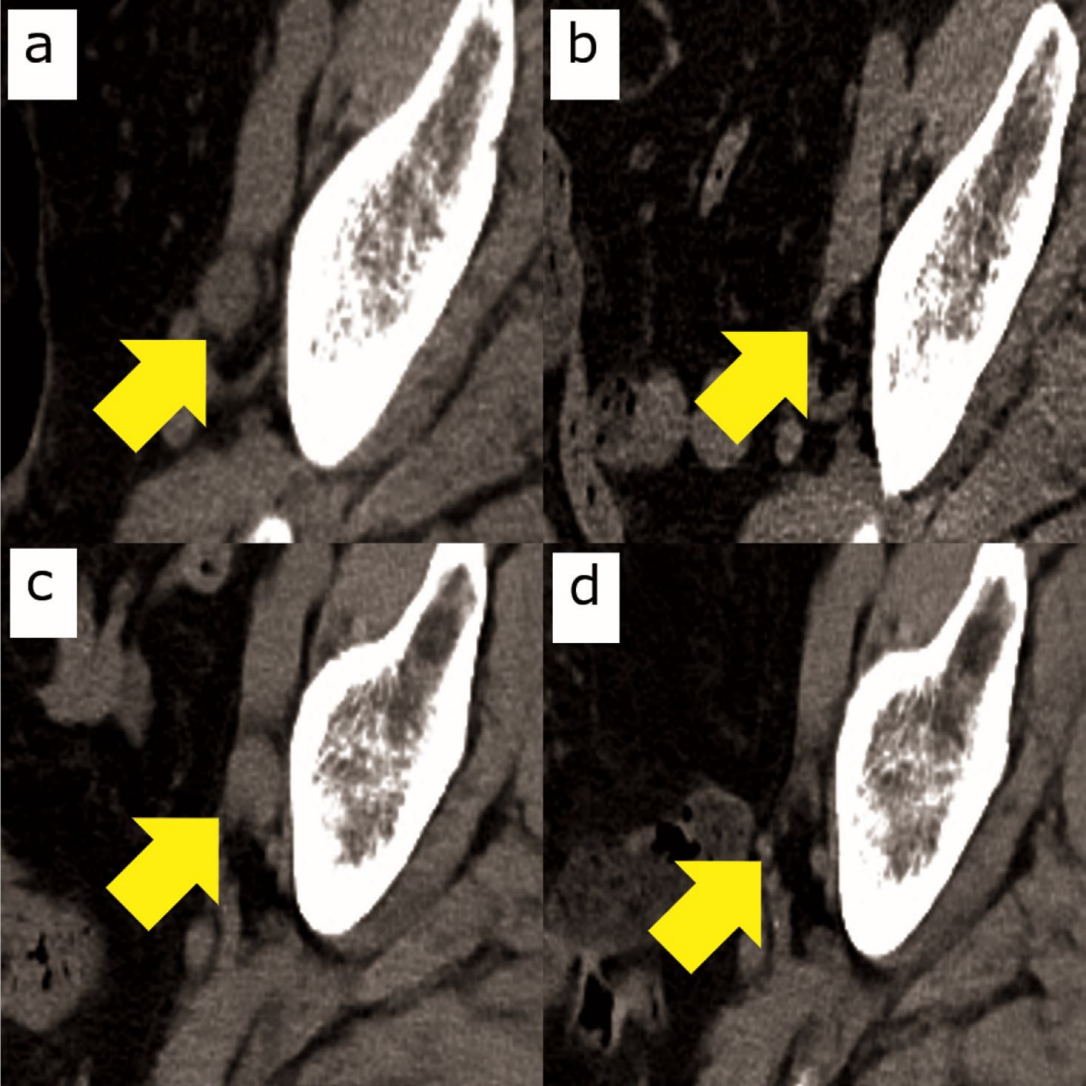
Radiologic course demonstrating recurrence, progression on avelumab maintenance, and complete response to pembrolizumab. Sequential computed tomography (CT) images of the same left obturator lymph node show: all images acquired at comparable axial level and window settings. (a) Recurrent enlargement before gemcitabine–cisplatin chemotherapy (short‐axis 15 mm). (b) Complete response after chemotherapy (< 10 mm). (c) Regrowth after 16 cycles of avelumab maintenance indicating progression (short‐axis 12 mm). (d) Disappearance of the lesion after four cycles of pembrolizumab, confirming a second complete response (< 10 mm).

**FIGURE 2:**
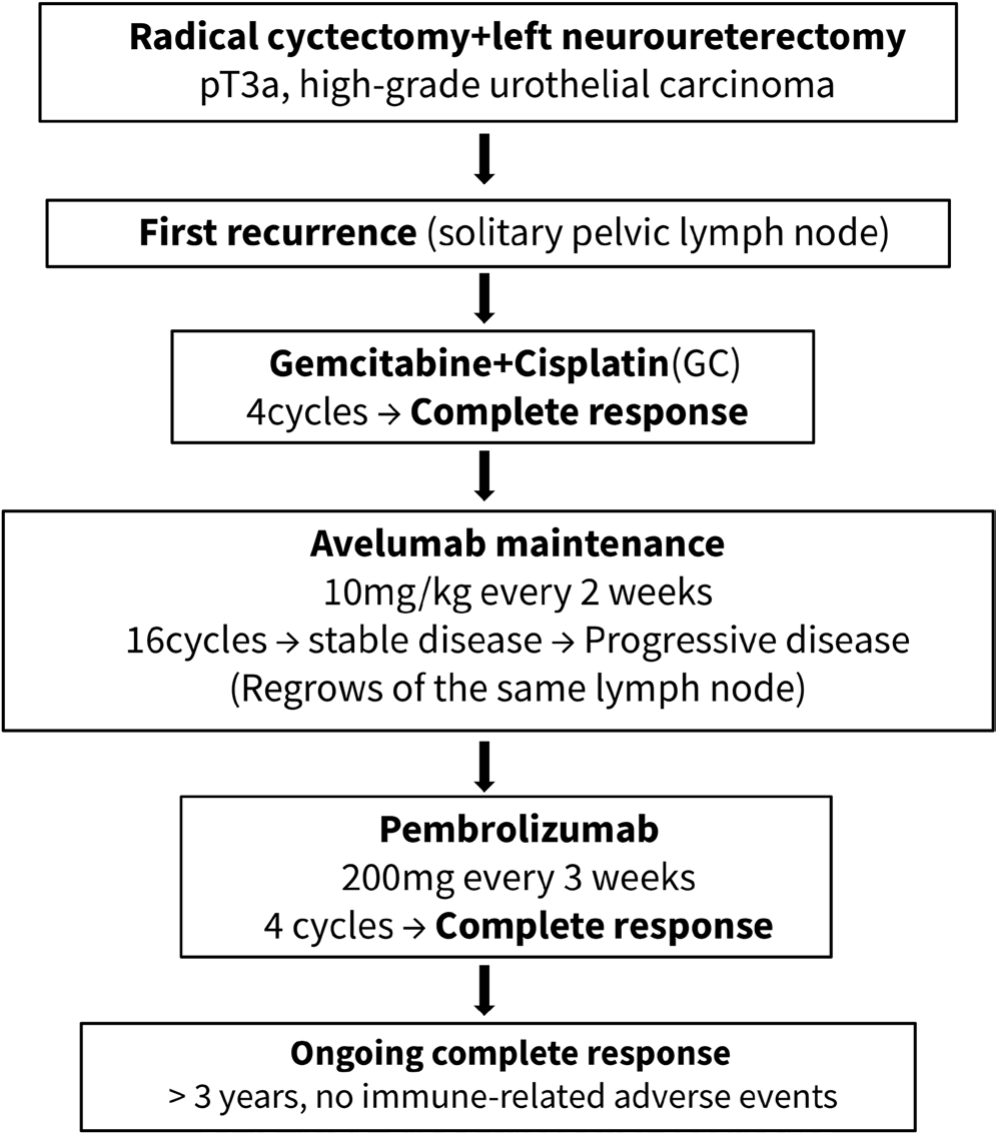
Clinical course and treatment timeline. The figure illustrates the sequential treatments and clinical responses in this patient. Following radical cystectomy and left nephroureterectomy for pT3a high‐grade urothelial carcinoma, the patient developed a solitary pelvic lymph node recurrence. Complete response was achieved with gemcitabine plus cisplatin chemotherapy. Disease progression subsequently occurred during avelumab maintenance therapy. Pembrolizumab was then administered, resulting in a durable complete response that has been maintained for more than three years without immune‐related adverse events.

**FIGURE 3:**
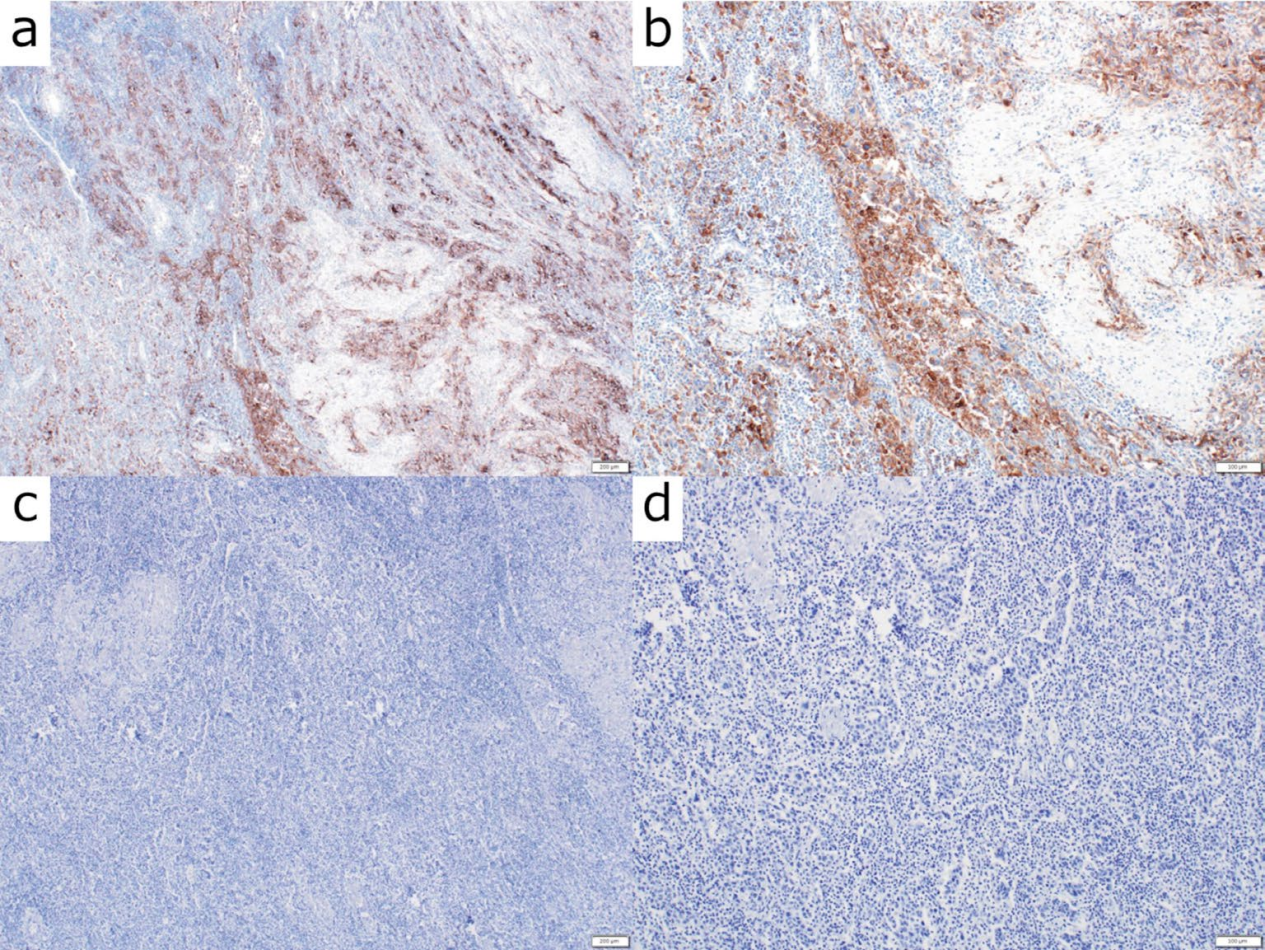
Immunohistochemical findings of the primary tumor. Representative immunohistochemical staining of the primary urothelial carcinoma obtained from the radical cystectomy specimen. (a) Low‐power view (40×) of PD‐L1 immunostaining (clone 22C3) demonstrating heterogeneous positive expression within the tumor. (b) High‐power view (100×) of PD‐L1 immunostaining showing membranous staining of tumor cells, with a combined positive score (CPS) of 20%. (c) Low‐power view (40×) of PD‐1 immunostaining showing minimal expression in the tumor microenvironment. (d) High‐power view (100×) confirming the absence of significant PD‐1–positive immune cells (CPS 0%). All images were obtained from formalin‐fixed, paraffin‐embedded tissue sections. Scale bars: 200 μm in (a, c); 100 μm in (b, d).

Pathological findings (radical cystectomy specimen): high‐grade urothelial carcinoma, pT3aN0M0; lymphovascular invasion absent; surgical margins negative; no variant histology identified. Surveillance computed tomography (CT) two and a half years later revealed a solitary enlarged left obturator lymph node consistent with recurrence (short‐axis diameter: 15 mm). At recurrence, his ECOG performance status was 0 and baseline eGFR was 58 mL/min/1.73 m^2^. Four cycles of gemcitabine plus cisplatin (cisplatin dose reduced to 75% for renal function) achieved radiographic complete response (the lymph node regressed to < 10 mm). Avelumab maintenance (10 mg/kg every two weeks for 16 cycles) was initiated. After 16 cycles, interval CT demonstrated regrowth of the same nodal lesion, confirming progression (short‐axis diameter: 12 mm). Avelumab was discontinued and pembrolizumab (200 mg every three weeks) was started. At that time, pembrolizumab use after avelumab maintenance failure was off‐label in Japan. In line with our institutional policy for off‐label use in individual patients, a multidisciplinary discussion was held and written informed consent for off‐label treatment was obtained from the patient. After four cycles, CT showed a second complete response (< 10 mm). Pembrolizumab has been continued; the patient remains disease‐free three years after pembrolizumab initiation and seven years after cystectomy, with no immune‐related adverse events. The treatment timeline and imaging are summarized in Figures 1 and 2, respectively. Immunohistochemical analysis was performed on archival formalin‐fixed, paraffin‐embedded tissue from the primary radical cystectomy specimen. PD‐L1 (22C3) demonstrated a combined positive score (CPS) of 20%. PD‐1 staining was negative (CPS 0%). Metastatic tissue was not available for immunohistochemical analysis (Figure 3).

## 3 Discussion

At the time of progression on avelumab maintenance in mid‐2021, enfortumab vedotin had not yet been approved or reimbursed in Japan for post‐platinum and post–immune checkpoint inhibitor disease. Therefore, pembrolizumab monotherapy was selected as an off‐label option after institutional oversight and written informed consent. Since then, emerging Japanese real‐world evidence has begun to clarify the outcomes of enfortumab vedotin following avelumab maintenance failure. A recent multicenter retrospective study from Japan reported clinical outcomes of enfortumab vedotin in this setting, supporting enfortumab vedotin as the preferred treatment option after progression on avelumab maintenance in current practice [8]. Nevertheless, our case suggests that switching from PD‐L1 to PD‐1 blockade may remain a reasonable alternative in carefully selected patients with oligometastatic, slowly progressive disease and low tumor burden, particularly when antibody–drug conjugates are unavailable or unsuitable.

This case illustrates a durable remission after switching from PD‐L1 to PD‐1 blockade following progression on maintenance therapy. Avelumab maintenance improves survival after first‐line chemotherapy [1, 3]. When progression occurs, enfortumab vedotin provides a proven survival benefit after prior platinum and an immune checkpoint inhibitor [4], and the combination of enfortumab vedotin plus pembrolizumab is the new first‐line standard [5]. However, for patients relapsing specifically after avelumab maintenance, evidence supporting either EV monotherapy or EV–pembrolizumab combination remains limited. In indolent or oligometastatic relapse with low tumor burden, preserved organ function, or contraindications to antibody–drug conjugates, re‐challenge with PD‐1 blockade may be reasonable even in the modern era.

Baseline tumor burden and metastatic pattern have been reported to influence the efficacy of immune checkpoint inhibitors in metastatic urothelial carcinoma, supporting the clinical plausibility that low‐volume, indolent disease may derive sustained benefit from PD‐1 blockade [9].

Despite PD‐L1 positivity (CPS 20%), the tumor progressed during avelumab maintenance but achieved a durable complete response to pembrolizumab. This finding supports a biological distinction between PD‐1 and PD‐L1 blockade. Unlike PD‐L1 antibodies, PD‐1 inhibitors block interactions with both PD‐L1 and PD‐L2, whereas PD‐L1 antibodies leave PD‐L2–mediated signaling intact and may more effectively reinvigorate exhausted T cells even after apparent resistance to PD‐L1 inhibition [7, 10, 11, 12, 13].

The present case suggests that PD‐1 blockade can retain antitumor activity in selected patients with preserved immunogenicity, low tumor burden, and indolent disease kinetics, even after progression on PD‐L1 maintenance therapy. Together with emerging evidence on immune checkpoint inhibitor rechallenge across multiple malignancies, including metastatic urothelial carcinoma as well as lung cancer and melanoma, this observation supports biomarker‐ and disease‐kinetics–guided consideration of ICI rechallenge strategies [7, 14, 15, 16, 17, 18].

Consistent with this observation, a recent Japanese case report described avelumab‐resistant upper urothelial carcinoma responding to subsequent pembrolizumab therapy, further supporting the potential for PD‐1 blockade to overcome resistance to PD‐L1 inhibition in selected patients [18].

In current practice, PD‐1 rechallenge may still be appropriate for slowly progressive, oligometastatic disease with favorable host–tumor dynamics, whereas EV‐based therapy remains preferred for rapidly progressive disease.

Another unresolved issue is the optimal duration of PD‐1 blockade after a long‐lasting complete response. While pembrolizumab has been continued in the present case, several pivotal trials of PD‐1/PD‐L1 inhibitors incorporated predefined treatment durations (e.g., treatment discontinuation at approximately 2 years) and suggested that cessation after sustained response may be feasible in carefully selected patients. In the absence of prospective data specifically addressing post‐avelumab maintenance failure, treatment duration should be individualized based on response durability, patient preference, and the risk of immune‐related adverse events.

The reproducible depth and duration of response observed in this case suggest that a subset of tumors may retain functional immune responsiveness after PD‐L1 inhibition, which could potentially be leveraged through biomarker‐guided sequencing strategies. Prospective validation incorporating PD‐L2 immunohistochemistry, immune gene expression profiling, and circulating T‐cell clonality analysis may help identify patients who can safely and effectively benefit from PD‐1 rechallenge after avelumab maintenance failure.

This report has several limitations. Immunohistochemical analysis was limited to the primary tumor specimen, as metastatic tissue was not available for evaluation. In addition, PD‐L2 immunohistochemistry could not be performed because this assay is not available in routine clinical pathology laboratories and is restricted to research settings in Japan. The single‐patient nature of this report and the off‐label use of pembrolizumab after avelumab maintenance failure may limit generalizability.

## 4 Conclusion

Pembrolizumab induced a durable complete response after progression during avelumab maintenance in metastatic urothelial carcinoma. Sequential use of PD‐L1 and PD‐1 inhibitors may overcome resistance in select patients and warrants prospective evaluation with biomarker‐driven selection—particularly incorporating PD‐L2.

## Ethics Statement

According to the policy of our institution, single‐patient case reports do not require formal IRB approval.

## Consent

Obtained from the patient and patient's family.

Off‐label Use: Pembrolizumab after avelumab maintenance failure constituted off‐label use in Japan. Per institutional policy for individual‐patient off‐label therapy, the case underwent multidisciplinary review, and written informed consent for off‐label treatment and for publication (including images) was obtained.

## Conflicts of Interest

The authors declare no conflicts of interest.

## Data Availability

Data sharing not applicable to this article as no datasets were generated or analysed during the current study.
